# Studying fencing lunge accuracy and response time in uncertain conditions with an innovative simulator

**DOI:** 10.1371/journal.pone.0218959

**Published:** 2019-07-09

**Authors:** Anthony Sorel, Pierre Plantard, Nicolas Bideau, Charles Pontonnier

**Affiliations:** 1 Univ Rennes, Inria, M2S - EA7470, F-35000 Rennes, France; 2 Univ Rennes, CNRS, Inria, IRISA - UMR 6074, F-35000 Rennes, France; Hochschule Trier, GERMANY

## Abstract

Lunge motion is one of the fundamental attacks used in modern fencing, asking for a high level of coordination, speed and accuracy to be efficient. The aim of the current paper was the assessment of fencer’s performance and response time in lunge attacks under uncertain conditions. For this study, an innovative fencing lunge simulator was designed. The performance of 11 regional to national-level fencers performing lunges in Fixed, Moving and Uncertain conditions was assessed. The results highlighted notably that **i)** Accuracy and success decreased significantly in Moving and Uncertain conditions with regard to Fixed ones **ii)** Movement and Reaction times were also affected by the experimental conditions **iii)** Different fencer profiles were distinguishable among subjects. In conclusion, the hypothesis that fencers may privilege an adaptation to the attack conditions and preserve accuracy instead of privileging quickness was supported by the results. Such simulators may be further used to analyze in more detail the motor control strategies of fencers through the measure and processing of biomechanical quantities and a wider range of fencing levels. It has also a great potential to be used as training device to improve fencer’s performance to adapt his attack to controlled opponent’s motion.

## Introduction

Fencing is a well-recognized Olympic Games sport [1]. This sport, asymmetric by nature, asks for a high level of coordination, explosive strength, speed and accuracy. The lunge motion is one of the core components to be mastered in fencing. It consists in an explosive extension of the front leg accompanying a classical attack -extension of the sword arm threatening the touch zone. The control mechanism behind the lunge performance has been largely explained and investigated at kinematical and muscle levels [2–5].

Moreover, the performance of a fencer highly depends on the quickness of his movements regarding the opponent’s action. The unpredictable nature of this opposition requires strong open skills [6]. The fencer needs to analyze and select visual information provided by his opponent before the beginning of his motion [7, 8]. Once the fencer clarify where the information must be taken from, he has to react readily. A good coordination is required to achieve both speed and accuracy. The reaction time and motion time of a fencer to perform simple responses after a visual stimuli was evaluated in several studies [9, 10]. Although quickness is a key factor to achieve a successful lunge facing an opponent, these temporal criteria do not seem to be affected by the level of fencers [11, 12]. However, the speed of the sword during the attack was identified as a performance criterion [13, 14]. Most of these studies and several others have been conducted in static situations, involving the pointing of a static target with different pointing conditions and measures [15–19]. There is a real need to understand the performance and the behavior of the fencer facing uncertainty. Therefore, the aim of the current study was the assessment of fencer’s performance and response time in lunge attacks under uncertain conditions. In particular, the following assumption was evaluated: fencers may adapt their gesture to keep a reaction capacity to an uncertain situation, privileging an adaptation to the attack conditions and preserve accuracy instead of privileging quickness. After a short review dealing with uncertainty in fencing lunge, the experimental protocol, including an innovative fencing lunge simulator are presented. Individual and global results of a cohort of 11 regional to national level fencers are then presented and discussed.

## Related work

Previous works [2, 10] proposed to include the concept of uncertainty as a stimulus perturbation during the performance evaluation of fencing attacks. Indeed, such a perturbation of the lunge motion is likely to appear in match situations and may reveal adaptive behaviors of interest to better understand the performance of the athletes. Existing training devices may be used to analyse fencer’s responses to uncertain or perturbed conditions. For instance, the Meteres and the Arvimex systems are instruments with lights, targets and a stopwatch, simulating different temporal and spatial uncertainty comparable to a real opponent behavior. By triggering lights to activate the different targets, this device, together with a photoelectric cell placed at the height of the fencer’s hand, allow to measure reaction time and movement time during different assault conditions. Using the Meteres, [11] showed that expert fencers were significantly faster than novice ones during motor phase but not during reaction phase, while [20] noted that changing target position during assault led to a significant increase of the response time, but not of the reaction time. Nevertheless, the fixed locations of the enlightened targets placed at discrete positions led to a limited set of valid motor responses. These responses can be learned, involving in essence closed motor skills.

Several studies have confirmed an increase in response-reaction time when uncertainty increases [10, 21, 22]. However, the patterns of movement do not appear to be affected by this uncertainty [21, 23]. Finally, the time at which the target change occurs was also evaluated, and shows that an early change of the target in the motion sequence does not affect reaction time, in contrast to late changes [24]. These recent works highlighted the impact of the visual perturbation in the performance of the fencer. However, several methodological points may be improved. First, the time of the target position’s change is relative to the individual timing performance, which leads to a standardization issue between subjects since the perturbation triggering was individualized. Second, in [21, 23], the system gave only a binary information of success/failure of the touch for each lunge. Therefore, it did not give any information about the accuracy of the lunge with regard to the experimental conditions, that is an important factor to analyze in order to understand the performance of the fencer. Last, the fixed position of the targets leads to the same bias as the work evoked before. This last limitation was decreasing the ecology of the situation, since the target to reach in real match situation are not so clearly defined in discrete positions, and can be considered as a lack of flexibility of the research protocol. Moreover, even in uncertain conditions where the task can change (offensive task to defensive one or target position change), the prior knowledge of the few fixed positions where the target could be located can lead to stereotyped responses of the fencers to the perturbations, which limits the interpretation of such a study. Furthermore, the concept of uncertainty proposed in those studies is unclear, meaning either that the stimulus may change or will change. From the subject’s point of view, it could be considered as two slightly different tasks involving different response inhibitions [25].

Therefore, performance and biomechanical analyses of fencing lunges in uncertain conditions may benefit from more complete simulation setups, enabling an involvement of the subject close to real situations, flexible enough to simulate a continuous variety of targets to reach, and able to provide quantitative assessments of performance and biomechanics. The following study proposes to assess performance and response times of fencers during lunge attacks under uncertain conditions thanks to a novel fencing lunge simulator enabling the analysis of parameterizable and variable attack situations. In particular, the following assumption, denoted **[H1]**, was evaluated: fencers may adapt their gesture to keep a reaction capacity to an uncertainty (the fact that a target may move or not), privileging an adaptation to the attack conditions and preserve accuracy instead of privileging quickness.

## Material and methods

To test the assumption **[H1]** (see Related Work), the following experiment has been conducted with a cohort of regional to national level male fencers: participants performed several -sword- fencing attacks to a movable target placed in front of them under different experimental conditions, as described in the Experimental Protocol. An innovative fencing lunge simulator, described below, has been especially designed for this purpose.

### Fencing simulator

**Fig 1:**
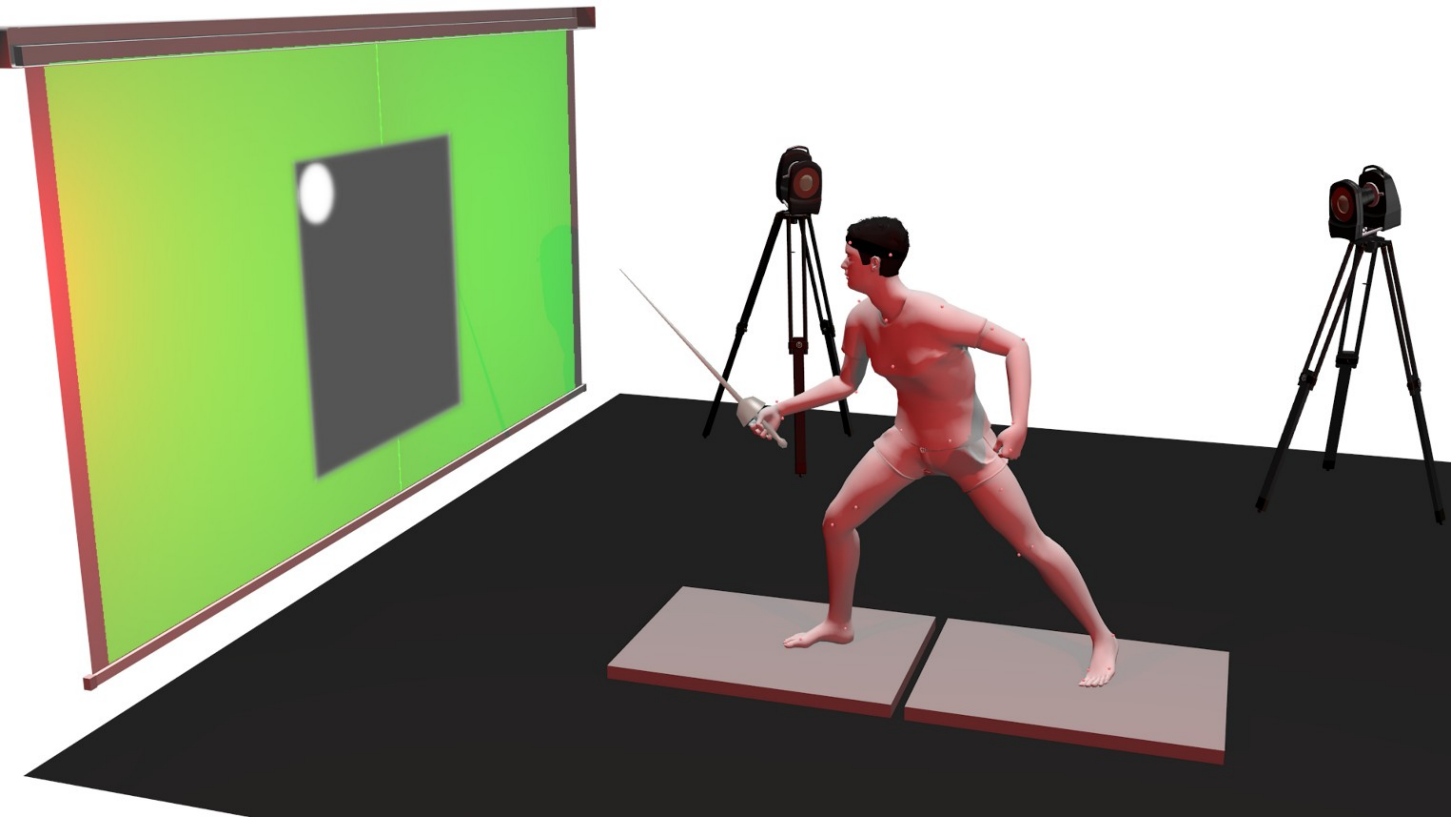
Experimental setup of the fencing lunge simulator. The fencer initially stoods on 2 platforms recording ground reaction forces. An optoelectronic system captured the movements of markers attached to the sword and the fencer. Target, plastron and surrounding color indicating the task to perform are back projected onto a wide screen of 5 x 2.2m. Green color here indicates the target will remain fixed during next attack.

The fencing simulator illustrated in Fig 1 consisted in an experimental device enabling a fencer to perform simulated fencing attacks on a virtual plastron representing the opponent and enabling the record of numerous data. The fencer placed his feet on two force platforms (AMTI, 120 x 60cm) embedded in the floor plane allowing to record ground reaction forces at 1000Hz during the motion. The target to touch was represented by a white circle into a dark rectangle mimicking the opponent’s plastron. Initial lunge distance, say distance between the screen and the fencer’s rear foot, was standardized as 1.5-fold the standing height of the fencer, as proposed by [3] for a long lunge. However, the fencer was free to slightly adapt his initial position to feel comfortable. Around the black rectangle, a color was also continuously projected 3 seconds before target appearance to indicate to the fencer which type of task he was currently performing (see Experimental Protocol). The target position was controlled by a software application allowing to vary its size and its initial and final positions in a standardized way (see Experimental Protocol). Such a software enabled the development of several application scenarios in a very flexible way.

**Fig 2:**
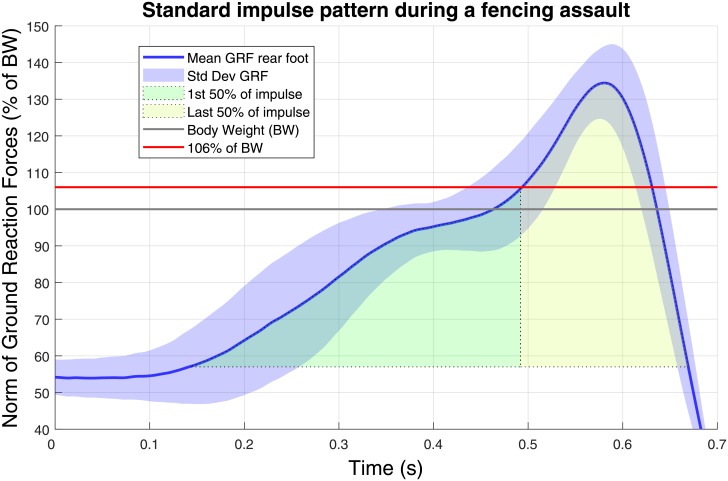
The ground reaction force threshold triggering the target motions was set so that half of the total impulse of a standard fencing assault had been produced. The mean ground reaction force norm leading to this condition corresponded to 106% of the subject body weight.

Previous works proposed either to move the target stimulus based on the release of a switch contact [10] or after a fixed personalized amount of time derived from prerecorded trials of the fencer [21], including his reaction time and movement time. In the current work the stimulus change was only based on the single body weight of the subject and this change could not occur until the fencer actually moves. Indeed, the target motion was automatically triggered by out-of-limit of the ground reaction force norm delivered in real-time by the force platform under the rear foot, relative to a predefined threshold corresponding to 106% of the body weight. This threshold has been defined by averaging the impulse pattern of ground reaction forces over 50 standard fencing assaults performed by a first cohort of 5 male fencers (3 right handed, 2 left handed, aged 22.5*y* ± 4.5*y*, height 1.76*m* ± 0.07*m*, weight 73*kg* ± 11*kg*) in a pilot experiment. This assault was considered as mechanically irreversible when at least 50% of its total impulse energy toward target has been produced by the fencer. At this point, his center of mass was engaged with a momentum that would require at least one step forward to maintain balance and the fencer was thus assumed to no longer be able to cancel his action. For real-time purpose this impulse threshold which was integrated over time had to be converted into an equivalent threshold on the ground reaction force norm which was time-independent. Fig 2 depicts how the threshold value was chosen.

**Fig 3:**
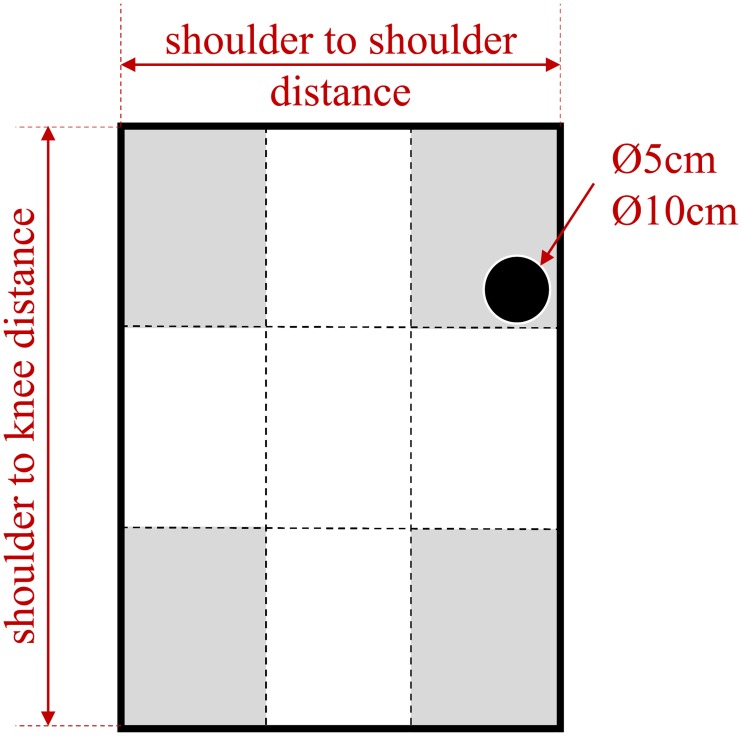
Plastron representation with target zones. Grey shaded areas represent the zones in which the targets could appear. The scenario only defined a target’s appearance zone, and the target appeared randomly in this zone. Plastron dimensions were computed with regard to the average height of a fencer. Shoulder to shoulder and shoulder to knee distances (0.26 x 0.82m) were computed from [26, 27]. The plastron height was adapted to be the same as the fencer’s one [23].

The initial and final positions of the target were randomly determined anywhere inside areas located in the four corners of the plastron as illustrated in Fig 3. These areas were much larger than the target size, preventing the target to appear always in the same position and thus the fencer to program his movement in advance. The required movement regulation that arose made the fencer facing an open-skill motor task.

A Plexiglas window protected the screen to allow the fencer to finish its attack. To ensure co-localization of the real and virtual elements of the experimental setup, position of the projected plastron and motion of the sword were captured thanks to a Vicon system (24 cameras, 250Hz sampling rate, 5.5*ms* latency) and reflective markers. 5 markers are placed around the screen and 7 markers on the sword as follows: 4 on the bowl, 3 on the blade (one at the very beginning next to the bowl, one in the middle and the last just under the tip). A Master PC was connected to the hardware acquisition system (Vicon Giganet) and ran both the recording (Vicon Nexus 2.5) and the rendering softwares (Unity3D). It also drove the video-projector (BenQ W1070, DLP technology, 120Hz, latency < 1 ms) that displayed the stimulus. In real time, Unity3D was listening Vicon Nexus through Vicon Datastream SDK to collect sword position and ground reaction forces under fencer’s feet that synchronously triggered changes of the target position as planned in the trial scenario. Such a system ensured a controlled latency of 35 ms between the acquisition and the final stimulus display. Effective touch on the plastron was determined by capturing the sword tip position at the impact on the Plexiglas window and was localized with regard to the target’s position.

### Participants

11 male fencers (5 right handed, 6 left handed, aged 22*y* ± 3*y*, height 1.78*m* ± 0.06*m*, weight 75*kg* ± 8*kg*) competing in épée (10 fencers) and foil (1 fencer) disciplines took part in the experiment after signing an informed consent form. 5 have participated in national competition, 5 in regional competition and 1 was a recreational fencer.

This study was approved by the INRIA national ethics committee (Comité Opérationnel d’Evaluation des Risques Légaux et Ethiques, COERLE notification number 2017-007).

### Experimental protocol

Prior to the experiment, a specific warm up of 15 min was carried out to let the fencer apprehend the simulator [2]. Each subject was equipped of a fencing helmet to be in situation. At the beginning of each experimental trial, the subject was informed the trial was about to start and the color corresponding to the trial condition was displayed (see next paragraph). After the subject noticed the experimental condition and confirmed he was ready, he received instructions to remain in an initial “en garde” position until the target appeared after a random amount of time between 0.5s and 1.2s as proposed by [12]. Then he executed a direct long lunge as quickly as possible to place the tip of the sword inside the target. The target’s zone of appearance was randomized to reduce learning effects.

Three experimental target motions were tested in this experimentation. During the preparation phase before the “en garde” position previous to the trial, the fencer was notified of the condition thanks to the color surrounding the plastron:

- **Fixed**: the target remains fixed during the attack (green surrounding color);
- **Moving**: the target will move during the attack (red);
- **Uncertain**: the target may move or not during the attack (blue). Leading to two sub-levels conditions: **Uncertain Fixed**—the target was eventually Fixed, and **Uncertain Moving**—the target was eventually Moving.

Two targets sizes were also tested (diameters of 0.05*m* and 0.10*m*). The global target zone was designed to correspond to the classical zone that can be reached in the épée discipline (Shoulder to shoulder and shoulder to knee distances), and its dimension was adapted thanks to an anthropometric table to correspond to an average fencer height of around 1.80m as reported by [26]. Fig 3 is describing the target zones and their dimensions. The vertical position of the plastron was adapted to the size of the subject, according to [23].

Each fencer performed a total of 120 attacks, divided in 4 sessions of 30 consecutive trials. Between each session, a break of 1 minute has been respected to avoid additional fatigue. Between each attack of a session, a delay of 10 seconds was respected. The target motion of the 120 attacks were randomly divided as follows: 16 Fixed target trials, say 4 repetitions for each 4 target zones; 44 Moving target trials, say 4 repetitions for each 4 initial zone moving toward 3 remaining final zones, except that trials where the sword arm may occult the target final zone were removed—from the upper controlateral side of the plastron (left for a right-handed fencer and conversely) to its lower lateral side (right for a right handed fencer and conversely); 16 uncertain Fixed targets and 44 uncertain Moving targets as described for the other conditions.

### Recordings and post-processing

**Fig 4:**
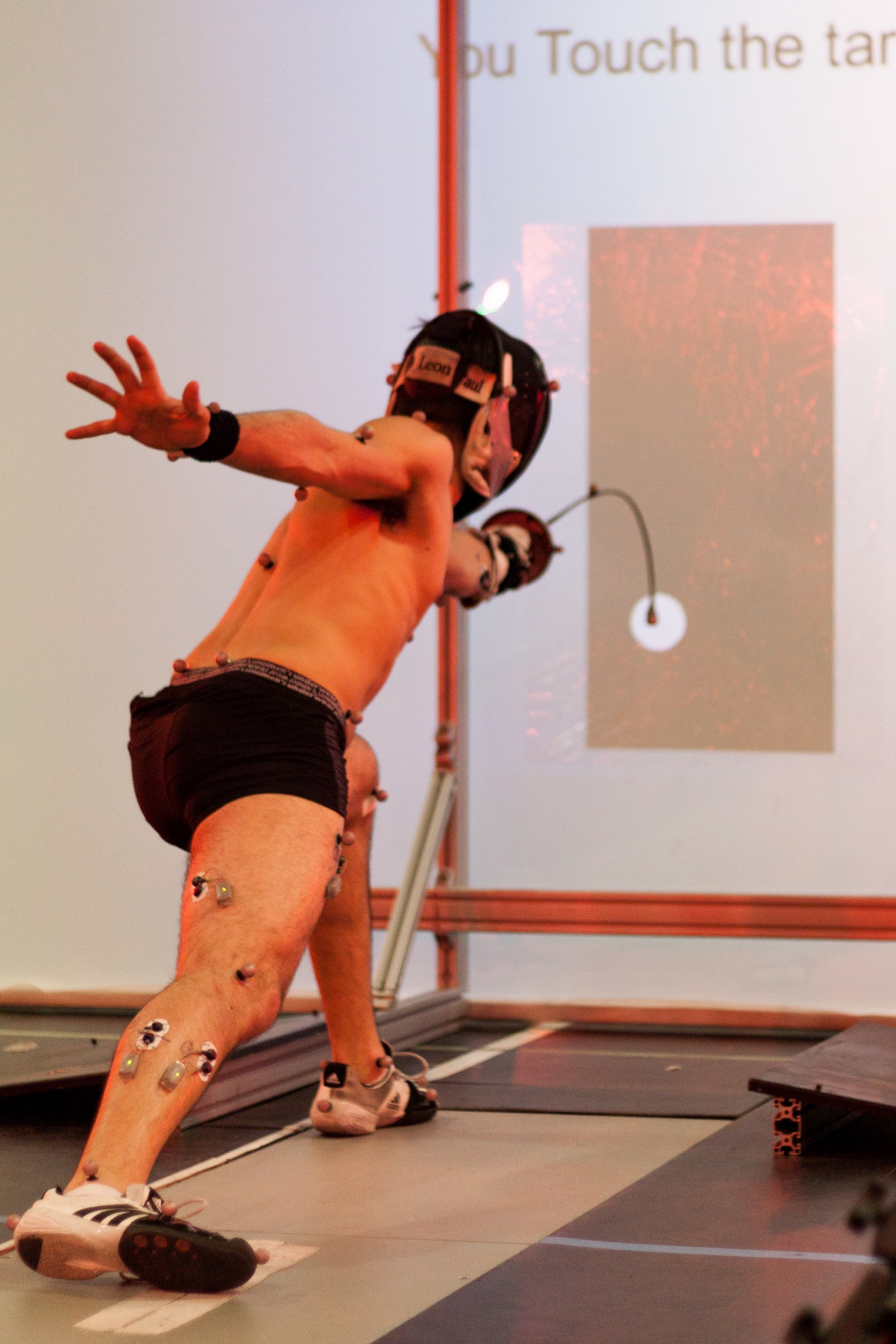
End of a successful lunge trial with a big target (0.10*m* diameter). Success information was displayed in real time by the system on the top of the plastron. Muscle activity and motion of the fencer were captured for further investigations.

Performance variables and timing variables—following definitions proposed by [2, 12], were computed from marker data and ground reaction forces:

- **Success**: percentage of successful attacks (attacks reaching the final position of the target);
- **Accuracy**: distance of the sword tip with respect to the target center at screen touch time, computed from markers positions. Expressed in *m*;
- **Reaction Time (RT)**: time between stimulus appearance and beginning of the fencer motion, defined as the moment when forward ground reaction forces exceeded 1% of the body weight, as specified in [28]. Expressed in *s*;
- **Movement Time (MT)**: time between motion beginning (after RT) and screen touch. Expressed in *s*;
- **Response Reaction Time (RRT = RT + MT)**: time between stimulus appearance and screen touch. Expressed in *s*;
- **Maximum Sword Velocity (MSV)**: maximum velocity under antero-posterior axis of the marker placed at the very beginning of the sword’s blade, next to the hand guard. Expressed in *m*.*s*^−1^;

For each attack, the system was able to compute these variable as soon as the sword hit the screen. Success was thus displayed on the screen in real time right after each trial as illustrated on Fig 4, however remaining information were hidden for the fencer to keep him focused on his task. Once the experiment was achieved, each fencer was immediately given a full report of his performance: success rate, accuracy, RT, MT, RRT for the overall experiment and also detailed performance by experimental condition and target size, as illustrated in S1 Video.

### Statistics

Mean performance of fencers were compared between different experimental conditions thanks to Wilcoxon signed-rank tests. First, multiple comparison were performed between Fixed, Moving and Uncertain target condition whatever the target size. Second, the target size’s effect (Small and Big) was investigated by considering all conditions pooled together. The confidence level was set to *p* < 0.05. Matched-Pairs Rank-Biserial correlations have been computed from [29]. Individual performance of the fencers were investigated through plots and mean performance analysis.

## Results and discussion

### Results

Success varied among participants from 80.8% to 21.7% (mean 53.3% ± 18.3%), whereas accuracy varied from 0.029*m* to 0.162*m* (mean 0.072*m* ± 0.039*m*). RT varied from 0.087*s* to 0.518*s* (mean 0.322*s* ± 0.120*s*), MT varied from 0.718*s* to 1.114*s* (mean 0.864*s* ± 0.114*s*) and RRT varied from 0.821*s* to 1.521*s* (mean 1.186*s* ± 0.183*s*).

**Fig 5:**
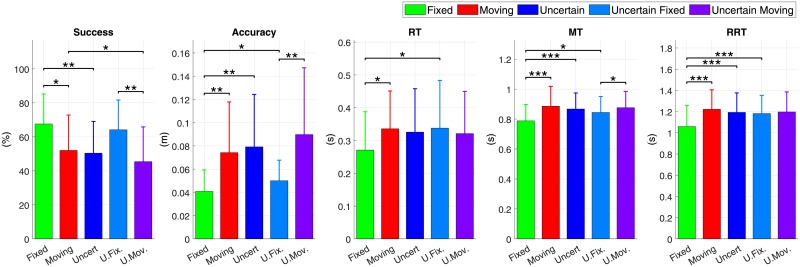
Overall mean results under the 3 different conditions (averaged by all the subjects). Significance of the Wilcoxon signed-rank test is expressed as follow: *** (*p* < 0.001); ** (*p* < 0.01); * (*p* < 0.05). NB: conditions Fixed/Uncertain Moving and Moving/Uncertain Fixed were not tested.

Fig 5 shows the average results of the subjects with regard to the experimental conditions. The statistical analysis revealed significant differences between observed variables under different experimental conditions. Table 1 provides exhaustive results on the evolution of the different variables when comparing the uncertainty conditions.

**Table 1:** Mean changes in the variable values (Shift), averaged for all the subjects, when comparing the different uncertainty conditions. For instance, the average Success rate was 15.5% lower in the Moving condition than in the Fixed one. Significance of the Wilcoxon signed-rank test is expressed as follow: *** (p < 0.001); ** (p < 0.01); * (p < 0.05). Matched-Pairs Rank-Biserial correlations (Rank Corr.) provides information on the effect size (a value above 0.8 indicates a large effect).

Variable	Compared conditions	Mean Shift	p	Rank Corr.
**Success** (%)	Fixed / Moving	-15.5 ± 15.4 (*)	.011	.833
	Fixed / Uncertain	-17.2 ± 13.9 (**)	.007	.879
	Moving / Uncertain	-1.6 ± 8.8	.717	.136
	U. Fixed / U. Moving	-18.8 ± 14.3 (**)	.002	.97
	Fixed / U. Fixed	-3.4 ± 18.6	.668	.318
	Moving / U. Moving	-6.6 ± 7.4 (*)	.02	.909
**Accuracy** (*m*)	Fixed / Moving	.033 ±.039 (**)	.01	.848
	Fixed / Uncertain	.038 ±.038 (**)	.002	.97
	Moving / Uncertain	.005 ±.011	.175	.485
	U. Fixed / U. Moving	.04 ±.049 (**)	.01	.848
	Fixed / U. Fixed	.009 ±.022 (*)	.042	.697
	Moving / U. Moving	.016 ±.02	.067	.636
**RT** (*s*)	Fixed / Moving	.065 ±.074 (*)	.019	.788
	Fixed / Uncertain	.055 ±.073	.102	.576
	Moving / Uncertain	-.01 ±.045	.465	.273
	U. Fixed / U. Moving	-.017 ±.037	.175	.485
	Fixed / U. Fixed	.067 ±.078 (*)	.024	.758
	Moving / U. Moving	-.015 ±.046	.32	.364
**MT** (*s*)	Fixed / Moving	.098 ±.049 (***)	.001	1
	Fixed / Uncertain	.08 ±.056 (***)	.001	1
	Moving / Uncertain	-.018 ±.052	.24	.424
	U. Fixed / U. Moving	.032 ±.04 (*)	.024	.758
	Fixed / U. Fixed	.056 ±.069 (*)	.019	.788
	Moving / U. Moving	-.01 ±.05	.52	.242
**RRT** (*s*)	Fixed / Moving	.163 ±.071 (***)	.001	1
	Fixed / Uncertain	.134 ±.066 (***)	.001	1
	Moving / Uncertain	-.029 ±.045	.067	.636
	U. Fixed / U. Moving	.015 ±.039	.206	.455
	Fixed / U. Fixed	.123 ±.055 (***)	.001	1
	Moving / U. Moving	-.025 ±.05	.102	.576
**MSV** (*m*.*s*^−1^)	Fixed / Moving	-.189 ±.218 (*)	.019	.788
	Fixed / Uncertain	-.186 ±.170 (**)	.007	.879
	Moving / Uncertain	.003 ±.070	.7	.152
	U. Fixed / U. Moving	-.084 ±.085 (**)	.007	.879
	Fixed / U. Fixed	-.124 ±.202	.067	.636
	Moving / U. Moving	-.019 ±.083	.175	.485

Success was higher in Fixed condition than in Uncertain condition (67.6% ± 17.6% vs 50.5% ± 18.6%) and higher in Uncertain Fixed condition than in Uncertain Moving condition (64.2% ± 17.5% vs 45.5% ± 20.4%). Accuracy was higher in Fixed condition than in Moving and Uncertain conditions (0.041*m* ± 0.018*m* vs 0.074*m* ± 0.044*m* and 0.079*m* ± 0.045*m* respectively). Accuracy was also higher in Uncertain Fixed condition than in Uncertain Moving condition (0.050*m* ± 0.018*m* vs 0.090*m* ± 0.057*m*). MT was lower in Fixed condition than in Moving and Uncertain conditions (0.788*s* ± 0.109*s* vs 0.886*s* ± 0.132*s* and 0.867*s* ± 0.107*s* respectively). Last, RRT was lower in Fixed condition than in Moving, Uncertain, and Uncertain Fixed conditions (1.059*s* ± 0.198*s* vs 1.222*s* ± 0.184*s*, 1.193*s* ± 0.183*s* and 1.182*s* ± 0.171*s* respectively).

MSV was significantly higher in Fixed condition (2.59*m*.*s*^−1^ ± 0.48*m*.*s*^−1^) than in Moving (2.41*m*.*s*^−1^ ± 0.38*m*.*s*^−1^) and Uncertain conditions (2.41*m*.*s*^−1^ ± 0.40*m*.*s*^−1^).

**Fig 6:**
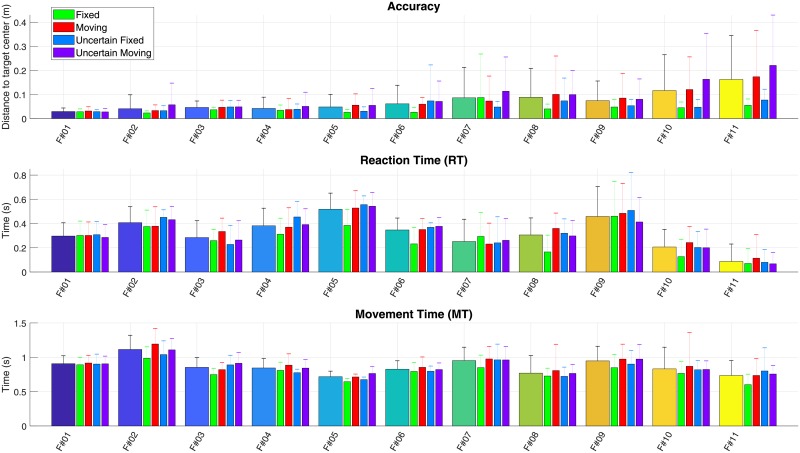
Individual results on accuracy (top), reaction time (middle) and movement time (bottom) under the 4 different conditions. Fencers are sorted according to their accuracy. For each fencer on x-axis, mean pooled values (large bars) are followed by 4 thin bars corresponding to Fixed, Moving, Uncertain Fixed and Uncertain Moving conditions (see legend). Both small and big targets have been taken into account here. Fencer #1 to #5 have participated in national competition, fencer #6 to #9 in regional competition, Fencer #10 was foil, Fencer #11 was recreational.

Fig 6 details the individual performances of each subject under each of the four conditions. As above-mentioned, it confirms that the majority of the subjects were negatively impacted in terms of Accuracy, RT and MT by Uncertain and Moving conditions compared to the Fixed one. In contrast, the best fencer seemed to be quite unaffected, as all his results sorted by condition were very close from his mean ones.

Target size effects were investigated and revealed a significant effect on success, higher for Big size targets than for Small size ones (72.2% ± 22.3% vs 42.3% ± 23.5%), and also on accuracy (0.060*m* ± 0.043*m* vs 0.067*m* ± 0.046*m*) and on MSV (2.49*m*.*s*^−1^ ± 0.41*m*.*s*^−1^ vs 2.44*m*.*s*^−1^ ± 0.41*m*.*s*^−1^), as reported in Table 2. No significant difference was found in any timing variables.

**Table 2:** Target size comparison. Mean changes in the variable values (Shift), averaged for all the subjects, when target size changes from Big (0.10*m*) to Small (0.05*m*). For example, success was 29.8% lower with small targets than with big ones. Significance of the Wilcoxon signed-rank test is expressed as follow: *** (p < 0.001); ** (p < 0.01); * (p < 0.05).

Variable	Shift	p	Rank Corr.
**Success** (%)	-29.8 ± 20.2 (***)	.001	1
**Accuracy** (*m*)	.007 ±.032 (*)	.024	0.758
**RT** (*s*)	.016 ±.062	.123	.545
**MT** (*s*)	.004 ±.066	.52	.242
**RRT** (*s*)	.020 ±.056	.067	.636
**MSV** (*m*.*s*^−1^)	-.045 ±.089 (**)	.007	.879

### Discussion

The current study proposed the evaluation of a cohort of regional to national level fencers in performing fencing lunges under uncertain conditions, thanks to an innovative fencing lunge simulator. Following sections are discussing the results with regard to the assumption **[H1]**.

#### Accuracy vs response time

Results of the study show relatively different profiles from one fencer to one other. Globally, the ability of the fencer to successfully and accurately touch the target seemed in contradiction with its ability to do it quickly. Levels of practice and fencer profiles have also to be considered to better understand the performance of the fencers in this study. For example, fencer #11 exhibited the lowest level of success and accuracy whereas he exhibited the best RT and RRT. Since the RT was lower than 100 *ms*, considered as the minimum time of physiological reaction [30], it is most likely that he anticipated largely the target appearance. He would therefore not have had enough observation time to adapt his attack to the possible motion of the target. All of the other fencers exhibited relatively similar performances in terms of RT, whereas their ability to touch the targets depended on their level of practice. Besides, the order of the fencers in Fig 6, classified from national level (#1 to #5) to regional (#6 to #10) and recreative (#11), illustrated the ability of the simulator to automatically retrieve the level of each participant by classifying them according to their accuracy.

#### Level of uncertainty

The level of uncertainty leads to several observations. First of all, under a full uncertain task, when no prior knowledge was provided on a possible target change, fencers exhibited a similar performance in terms of accuracy and overall success when the target remained finally fixed, as if they would have been aware of the Fixed condition. However, those performances significantly decreased when the target was finally Moving. When considering timing variables (RT, MT and RRT), fencers did not exhibit significant difference between full Uncertain and Moving condition. The only significant difference arised when the fencer knew for sure there won’t be any target motion (Fixed), confirming similar studies by [21, 22]. Results showed an increasing RT up to 24% and 20% when compared respectively to Moving and Uncertain conditions (however non significant for this latter condition), a significant increasing MT up to 12% and 10% respectively, leading to a significant total RRT increase of up to 15% and 12% respectively. So far, discrete choice reaction time has been quite studied and bio-informational approaches inherited from Hick’s work assumed that reaction time increases at a logarithmic rate with the number of stimulus-response alternatives [10, 31]. Uncertainty also lead to contradictory sensorimotor processes between activation and inhibition of motor responses [25, 32–35]. In light of these previous studies, the Fixed condition was dramatically reducing the number of valid motor alternatives to reach the target compared to Moving and Uncertain conditions, explaining the significant decrease in RRT under Fixed condition.

According to Table 1 most significant differences in terms of accuracy, success and timing arise from the comparison between Fixed and Moving, and Fixed and Uncertain trials (two first rows of each item). This is particularly visible seeing the third row (Moving/Uncertain comparison) that is never significant. These results show that fencers reacted to the Uncertain condition in a very similar way to the Moving one, and differently from the Fixed one. Such a result is consistent with **[H1]** since it indicates that participants preferred to ensure a good accuracy by slowing their motion rather than trying to be fast without accuracy. Individual results may mitigate this assertion due to the large dispersion of participants performance.

Hence, by comparison to [21, 22], in this study, timing variables (RT, MT and RRT) systematically showed greater values, regardless of the condition. In Fixed condition, RT was up to 40% longer. As opposed to these previous studies, where the few discrete possible positions of the target remained visible and unchanged, allowing to preprogram a full defined motor response for each target position, in this study, the target could appear continuously in wide corner areas of the plastron. As a result, even if four rough motor responses could be preprogrammed, a motor regulation had to be performed “online” to adjust the reaching movement to the exact final position of the target, requiring open skills. This motor regulation arised even in Fixed condition, as the target did not always appear in a predefined position, unlike previous studies.

Last, results of this study revealed a significant lengthening of the MT under Moving and Uncertain conditions, contradicting [21] which reported a non significant increase of 2% of MT with uncertainty growing. A possible explanation lies in the different ways the stimulus was changed: either by waiting for a fixed subject-specific amount of time, which allowed the fencer to “cheat” the system by delaying his assault until the actual stimulus appears, or triggered by force-plates synchronized with the display system, which made it harder to cheat. To clarify this point, further investigations should be conducted on the GRF threshold that triggers target changes.

Results of this work were extracted by gathering all the trials by condition, regardless of their success, in order to study how the task uncertainty influences performance and timing variables. A complementary study could be conducted to observe specifically the influence of the task success on the timing variables in the different conditions, as in [10, 12, 22].

#### Motor adaptation

Statistical tests revealed that Big size targets generated a higher Success than Small size ones. A slightly better Accuracy (7*mm*) has also been reported in Table 2, but must be mitigated regarding the size of the marker positioning the touch (16*mm* diameter). Its location just below the tip of the sword may introduce an additional distance, corresponding approximately to its radius, from the actual touch position when the blade bends. On the other side, no timing variables were significantly different for the two conditions. This may reveal no particular adaptation of the strategy between both target sizes: subjects did not try to touch more efficiently the target but only its center in any conditions that leaded to different success results without strongly disrupting timing variables and Accuracy. These variables provide an interesting metrics indicating changes in motor control strategies with regard to experimental conditions [25]. One may have expected different RRT and accuracy results due to the difficulty, as it has been seen in several publications gathered in [1].

#### Applicability to training

This fencing simulator could be used both as an evaluation and a training tool. It has proven to be relevant in differentiating the level of fencers through their accuracy in hitting their targets. Moreover, as revealed in Fig 6, it demonstrated its ability to highlight different fencer’s profiles by providing numerous different conditions mimicking real competition situations. Size, initial and final positions and instant change of the target could be tuned and triggered in a continuous and standardized way, making the system appropriate at evaluating and training open skills. For instance, the target zone may be adapted to different weapons as well as different specific exercises—touching only a given zone, fencing a specific morphology… This flexibility offers many analysis and training opportunities to the fencer, such as simulating moving targets automatically adapted to the fencer’s movement. The evaluation report delivered right after the end of the evaluation allows to guide the fencer to a suitable training program.

Several improvements could be introduced to fine-tune the evaluation and enhance training sessions. For instance, the final report could detail performances for each target area of the plastron, online and offline feedback on accuracy and timing performances could be provided. Stimulus could also be manipulated to propose some training scenarios, based on probabilistic information on target position, size or motion, as proposed in [36], or by adding defensive tasks as in [22].

This simulator requires expensive acquisition equipment and a rather complex software and hardware implementation. To be used out of the lab, such a system must be affordable. A convenient solution may be to make use of a rugged touchscreen to display the stimulus and collect the touch time and position, connected to a pressure maps or sole that gathers ground reaction pressure under the back foot. This would lead to a degraded simulator with a minimal loss of functionality.

#### Limitations and perspectives

The fencing lunge simulator proposed in this paper may be of real interest in motor control analysis as well as for training applications, as proposed above. However, several shortcomings are asking for improvements in the future:

- The simulator does not enable to change the depth of the attack, whereas a significant part of the adaptation can be explained by the motion of the defender in the antero-posterior direction [7]. Such a simulator is complex to design since depth implies the use of virtual reality to be simulated. Indeed the scene become 3D instead of 2D in the current version of the simulator, which also makes it difficult to reproduce the essential sensation of the sword’s impact at touch time.
- The simulator was intentionally set up as a neutral representation of the plastron of the adversary. An investigation about the level of detail and animation of the simulated opponent may have an interest to increase the realism of the simulator and its implication on fencer’s performance [37].
- The relatively small number of fencers involved in this experiment (11) as well as their heterogeneous level do not allow to extract more significant statistics while several additional trends seem to emerge.
- We also gathered several additional data usable to analyze the motor strategies, such as anticipatory postural adjustements [13], and the biomechanical performance of the fencer in a deeper way (electromyography, motion capture, ground reaction forces). Pilot results, presented in [38] on a single subject, indicated minor changes in upper limb muscular strategy depending on the uncertainty condition. This result has to be investigated with the subjects of the current study to be validated.
- As mentioned above, discrete choice reaction time has been extensively studied to demonstrate a logarithmic increase of the reaction time with respect to the number of stimulus-response alternatives [31]. The experimental setup proposed here allows to investigate this relationship in the continuous case, for instance by observing the influence of the plastron’s size on the reaction time under Fixed conditions.

### Conclusion

The current study proposed an evaluation in terms of accuracy and response time of a cohort of fencers performing fencing lunges under uncertain conditions. For this purpose, an innovative fencing lunge simulator has been designed and presented, allowing to test and validate the assumption **[H1]**: fencers adapt their gesture to keep a reaction capacity to an uncertain situation (the fact that a target may move or not), privileging an adaptation to the attack conditions and preserve accuracy instead of privileging quickness. Particularly, participants were significantly slower in Uncertain Fixed condition—when the target may move but did not—than in Fixed condition with a similar level of success, indicating an adaptation of the fencer to the condition by privileging its accuracy over its quickness in such situations. The simulator is flexible enough to simulate a continuous variety of targets to reach, and able to provide quantitative assessments of performance and biomechanical quantities. It opens perspective for the analysis of numerous and tunable attack situations under controlled and flexible conditions. In the near future, such a simulator may be used to analyze more in detail the motor control strategies of fencers through the measure and processing of biomechanical quantities [39] and a wider range of fencing levels. It has also a great potential to be used as training device to improve fencer’s performance to adapt his attack to the opponent’s motion in a degraded and more affordable setup.

## Supporting information

S1 Video Video illustration of the fencing simulator in experimental condition. (MP4)
